# Research progress on continuous nursing care for children after radical surgery for Hirschsprung’s disease

**DOI:** 10.3389/fpubh.2026.1913893

**Published:** 2026-08-27

**Authors:** Dezhi Lu, Qingyu Xu, Xiaohan Yu, Yi Song, Honglian Xia, Yufeng Li

**Affiliations:** 1 Department of Pediatric Surgery, Hong Qi Hospital Affiliated to Mudanjiang Medical University, Mudanjiang, Heilongjiang, China; 2 School of Nursing, Mudanjiang Medical University, Mudanjiang, Heilongjiang, China; 3 First School of Clinical Medicine, Mudanjiang Medical University, Mudanjiang, Heilongjiang, China; 4 Graduate School of Mudanjiang Medical University, Mudanjiang, Heilongjiang, China; 5 Surgical Anesthesia Center, Hong Qi Hospital Affiliated to Mudanjiang Medical University, Mudanjiang, Heilongjiang, China

**Keywords:** continuous nursing care, Hirschsprung’s disease, pediatric surgery, postoperative rehabilitation, radical surgery

## Abstract

Hirschsprung’s disease is a common congenital gastrointestinal malformation in children, for which radical surgery remains the primary treatment modality. Postoperatively, patients are prone to complications such as Hirschsprung-associated enterocolitis, defecation dysfunction, anal stenosis, and psychological or behavioral abnormalities, all of which significantly impair growth, development, and quality of life. As a critical bridge linking inpatient treatment with home-based rehabilitation, continuous nursing care has demonstrated efficacy in enhancing post-discharge outcomes, reducing complication risks, and improving long-term prognosis. This review systematically synthesizes the extant literature concerning continuity of care following radical surgery for pediatric Hirschsprung disease, both domestically and internationally. It delineates the core constructs, clinical significance, prevailing care models, key intervention strategies, and outcome evaluation frameworks within this domain, while concurrently summarizing current research deficiencies and projecting future developmental trajectories. The paper critically appraises recent advances in novel modalities—specifically informatized smart nursing, individualized precision care, and multidisciplinary collaborative care—and consolidates optimization outcomes related to core interventions, including bowel rehabilitation, complication surveillance, empowerment of family caregivers, and psychosocial support. Addressing identified gaps, this review proposes strategic directions encompassing the establishment of standardized nursing protocols, deeper integration of intelligent technologies, implementation of long-term prognostic surveillance, and deployment of precision interventions for high-risk pediatric subgroups. Notably, parental educational level is identified as a critical confounding variable exerting significant influence on home-care quality and rehabilitation efficacy; consequently, subsequent investigations should incorporate this metric into stratified analyses and nursing quality control systems. This synthesis aims to provide an evidence-based foundation for the standardized implementation of postoperative continuity of care and the optimization of rehabilitation management pathways, thereby propelling the evolution of pediatric surgical nursing toward enhanced standardization, precision, and intelligence.

## 1 Introduction

Hirschsprung’s disease (HD) is characterized by the absence of ganglion cells in the distal intestine, resulting from arrested neural crest cell migration during embryogenesis, which leads to intestinal dysmotility (1). The incidence of HD is approximately 1 in 5,000 live births, with a male-to-female ratio of about 4:1. Typical clinical manifestations include intractable constipation and abdominal distension, which may progress to life-threatening complications such as Hirschsprung-associated enterocolitis (HAEC) and intestinal perforation (2, 3). Laparoscopic-assisted transanal pull-through has become the mainstream curative procedure, effectively correcting anatomical anomalies; however, it does not immediately restore intestinal peristalsis or sphincter function, necessitating a prolonged postoperative recovery period (4). Moreover, most caregivers lack specialized home-care skills, often leading to suboptimal practices, interrupted rehabilitation, and delayed follow-up. Persistent defecation disorders may further contribute to psychological and behavioral disturbances, thereby impeding physical, emotional, and social development in affected children (5).

Continuous nursing care represents a systematic, cross-setting model that extends inpatient services into home and community settings. By integrating structured follow-up, rehabilitation guidance, risk monitoring, psychosocial support, and caregiver training, this model facilitates closed-loop management throughout the entire postoperative recovery continuum (6). With the growing adoption of enhanced recovery after surgery (ERAS) principles, precision nursing, and internet-based healthcare, continuous nursing strategies for pediatric HD have evolved considerably, demonstrating notable benefits in reducing complications, strengthening caregiver competence, and improving long-term outcomes. Nevertheless, current research efforts—both domestically and internationally—remain predominantly focused on perioperative in-hospital care, with limited evidence addressing long-term, post-discharge continuity of care. To address this gap, the present review synthesizes recent findings from the literature to delineate the current state and developmental trajectory of continuous nursing following radical surgery for HD, aiming to inform clinical practice and advance the specialization of pediatric surgical nursing.

This article presents a narrative review. Literature retrieval spanned from the inception of each database to June 2026, covering international databases (PubMed, Web of Science, Embase, Cochrane Library) and Chinese databases (CNKI, Wanfang Data, VIP). The search strategy combined the following English keywords: (“Hirschsprung disease” OR “congenital aganglionic megacolon”) AND (“continuity of care” OR “transitional care” OR “postoperative nursing” OR “home care” OR “follow-up care”) AND (“pediatric” OR “child”). The Chinese search terms were: (“先天性巨结肠”OR “无神经节细胞症”) AND (“延续性护理” OR “延续护理” OR “过渡期护理” OR “术后护理” OR “居家护理”). Inclusion criteria were: (1) studies involving pediatric patients after radical surgery for Hirschsprung disease; (2) studies focusing on postoperative continuity of care models, intervention strategies, or outcome evaluations; (3) literature types including original research articles, systematic reviews, clinical guidelines, expert consensus statements, and high-quality reviews; and (4) publications in English or Chinese. Exclusion criteria comprised: (1) duplicate publications; (2) conference abstracts or articles for which the full text was inaccessible; and (3) publications in languages other than English or Chinese. Following the selection process, a total of 97 articles were included in the final analysis, consisting of 68 English articles and 29 Chinese articles.

## 2 Core concept and clinical value of continuous nursing care after radical surgery for pediatric HD

### 2.1 Core concept

Continuous nursing care following radical surgery for pediatric HD centers on the child’s needs across the entire recovery continuum. Leveraging a tertiary linkage system encompassing hospital, community, and home settings, this model extends standardized in-hospital care into the post-discharge environment (7), spanning the short-term recovery phase, intermediate functional restoration phase, and long-term follow-up phase (Table 1). The scope of services encompasses multidimensional domains, including bowel function rehabilitation, complication surveillance, caregiver capacity building, and psychosocial-behavioral interventions. This evolution marks a paradigm shift from unimodal disease-specific nursing toward comprehensive, life-cycle health management (8, 9).

**Table 1:** Three-phase transitional care program following surgery.

Phase	Timeframe	Core components of care	References
Early recovery phase	1–3 months postoperatively	Basic disease monitoring, wound care, standardized anal dilation, early screening for complications, and fundamental training for home-based care.	(92)
Mid-term functional rehabilitation phase	3–12 months postoperatively	Bowel function training, defecation habit correction, dietary and nutritional management, preliminary psychological intervention, and regular functional assessments.	(56)
Long-term prognostic follow-up phase	≥12 months postoperatively	Monitoring for long-term complications, growth and development surveillance, cultivation of social adaptability, and maintenance of lifelong health records.	(93)

Grounded in theoretical frameworks such as Noddings’ Care Ethics (10), ERAS principles (11), and precision nursing (12), the model moves beyond traditional, homogeneous service delivery. Instead, it emphasizes individualized, phased care plans tailored to specific patient factors—including surgical approach, length of aganglionic segment, age, physical condition, and family caregiving capacity—thereby ensuring that nursing interventions are both scientifically sound and contextually appropriate.

### 2.2 Clinical application value

#### 2.2.1 Reducing postoperative complications and ensuring safe recovery

Postoperative long-term complications of HD, including HAEC, anal stenosis, fecal incontinence, and intestinal obstruction (13), predominantly manifest during the home-recovery phase. These conditions often present with insidious early symptoms; delayed intervention may precipitate severe sequelae such as overwhelming infection and intestinal failure. Standardized continuity of care facilitates the early identification and timely management of complications through structured follow-up schedules, systematic home-based symptom monitoring, and targeted caregiver risk education. This proactive approach effectively reduces the incidence of postoperative complications, decreases hospital readmission rates, and alleviates healthcare expenditures (8). Furthermore, sustained functional training regimens enhance anal sphincter integrity and optimize intestinal peristalsis, thereby mitigating functional abnormalities including constipation and diarrhea (14).

#### 2.2.2 Enhancing family caregiving capacity and standardizing home-based rehabilitation

Home rehabilitation after HD surgery imposes high demands on caregivers’ professional competencies. Procedures such as anal dilation, rectal irrigation, bowel training, dietary regulation, and medication management require specialized skills. The lack of systematic training among most caregivers often leads to operational errors and poor adherence, directly compromising recovery outcomes (15). Transitional care addresses this gap through preoperative specialized training, post-discharge one-on-one tele-guidance, distribution of illustrated educational manuals (16), online health education, and dissemination of instructional videos. These strategies enable caregivers to systematically acquire core home-care skills, correct inappropriate practices, and improve both the professionalism and compliance of family caregiving, thereby establishing an efficient rehabilitation model characterized by expert hospital guidance and precise home implementation (17). Parental educational level constitutes a critical determinant of proficiency in acquiring caregiving skills. Parents with lower educational attainment often encounter difficulties comprehending printed educational materials and navigating online platforms, rendering them prone to suboptimal practices such as non-standardized anal dilation techniques and delayed recognition of complications. Conversely, while highly educated parents generally demonstrate superior health literacy and information retrieval capabilities, enabling rapid mastery of standardized protocols, they face the potential risk of sourcing unreliable information from non-authoritative websites, which may lead to inconsistencies in care regimens (18). Consequently, caregiver guidance must be tailored to align with parental cognitive levels. Implementing multimodal educational strategies—integrating illustrations, video demonstrations, and hands-on practice—is imperative to mitigate disparities in care quality attributable to educational background.

#### 2.2.3 Improving physical and psychological wellbeing and optimizing long-term quality of life

Chronic defecation dysfunction often results in malnutrition and growth retardation in children with HD. Additionally, persistent physical discomfort and recurrent episodes predispose patients to psychological comorbidities—including low self-esteem, anxiety, social withdrawal, and isolation—which impair normal functioning and social adaptability (19, 20). Transitional care adopts a multidimensional approach, integrating physical rehabilitation with psychological support, growth surveillance, and social adaptation interventions. Through psychological counseling, behavioral guidance, dynamic monitoring of growth parameters, and individualized nutritional support, this model effectively ameliorates the overall health status of patients, promotes physical development, fosters positive social engagement, and ultimately elevates long-term quality of life (21).

#### 2.2.4 Refining the holistic rehabilitation system and promoting nursing homogenization

Transitional care bridges the discontinuity between inpatient treatment and home rehabilitation, establishing a closed-loop framework comprising “in-hospital precision nursing–discharge transition guidance–home-based continuous intervention–long-term follow-up assessment” (22). Moreover, standardized and protocolized transitional care procedures harmonize service benchmarks across hospitals of varying levels and among nursing staff with diverse expertise. This minimizes practice variations, advances the standardization of postoperative HD nursing services, and provides a robust foundation for the continuous improvement of clinical nursing quality.

## 3 Mainstream models and research progress of transitional care following radical surgery for pediatric Hirschsprung disease

### 3.1 Traditional follow-up model of transitional care

Traditional continuity-of-care models—centered on telephone follow-ups, outpatient clinic visits, and home visits—remain the most fundamental approaches in clinical practice (23). The core protocol involves scheduled postoperative telephone calls by the primary responsible nurse to assess recovery progress, reinforce adherence to outpatient review schedules, and address caregiver inquiries. While operationally straightforward and cost-effective, this model fulfills only baseline requirements for condition monitoring and health education. Consequently, it is primarily applicable to postoperative management in primary care settings and among pediatric patients categorized as low-risk or presenting with mild manifestations (24).

However, this paradigm exhibits notable limitations. Its reliance on standardized follow-up content precludes the delivery of personalized intervention strategies. Exclusive dependence on audio-based communication impedes the direct visualization of caregiving practices in the home environment, thereby compromising the timely identification of occult care-related risks. Furthermore, irregular scheduling and fragmented content undermine the implementation of structured, longitudinal rehabilitation planning, frequently resulting in missed follow-ups and delayed interventions (25). The absence of sustained, bidirectional communication channels fosters a passive receipt of guidance among caregivers, limits avenues for proactive consultation, and ultimately hinders the maintenance of treatment adherence (26). Therefore, while traditional models may serve as a foundational adjunct, they are increasingly insufficient to meet contemporary clinical demands for precision-driven, high-quality rehabilitative nursing.

### 3.2 Informatized and intelligent transitional care model

Against the backdrop of “Internet + Healthcare,” the informatized and intelligent transitional care model—leveraging new media and smart medical platforms—has emerged as a prominent research focus and represents the mainstream innovative trajectory for post-HD transitional care (27). Anchored on platforms such as WeChat official accounts, exclusive doctor–family WeChat groups (28), online follow-up systems, intelligent rehabilitation applications (apps), and teleconferencing systems, this model establishes a round-the-clock virtual nursing service framework (29, 30). It facilitates the standardization and normalization of rehabilitation guidance, disease monitoring, clinician–caregiver interaction, health education, and data tracking. Mature clinical applications of this model currently include: (1) Routine health education: Regular dissemination of multimedia resources (e.g., illustrations and videos) covering postoperative rehabilitation knowledge, nursing techniques, dietary restrictions, complication recognition, and emergency management, thereby supporting flexible, “micro-learning” by caregivers (31). (2) Real-time clinician–caregiver interaction: Caregivers can upload real-world data—such as defecation logs, wound images, and rehabilitation training videos—enabling nurses to provide targeted feedback and promptly correct improper practices. (3) Smart follow-up reminders: Automated, stage-specific alerts for clinical review, rehabilitation exercises, anal dilation, and vaccinations are pushed to families via the system, minimizing oversight (32). (4) Dynamic digital archiving: Follow-up outcomes, rehabilitation metrics, and complication profiles are entered into a centralized electronic rehabilitation record in real time, providing an evidence base for individualized care plan adjustments (33).

Existing evidence demonstrates that informatized smart nursing significantly shortens the postoperative recovery period for bowel function in pediatric patients and markedly reduces the incidence of complications compared with conventional follow-up models (34). Recent investigations have further integrated smart wearable devices to enable dynamic, precision monitoring of convalescence by tracking parameters such as intestinal peristalsis, defecation frequency, sleep patterns, and growth metrics. This evolution represents a paradigm shift from reactive to proactive care and constitutes a pivotal trajectory for the future development of intelligent nursing (29, 35). However, this model entails considerable barriers to implementation, requiring caregivers to possess smartphone proficiency and maintain stable internet connectivity. Parental educational level directly modulates the acceptance and efficacy of digital platforms; caregivers with lower educational attainment frequently exhibit digital illiteracy, struggling to comprehend application interfaces and operational workflows. This results in substantially diminished adherence to online nursing interventions. Such disparities are particularly pronounced in remote regions and among households reliant on older adult caregivers, thereby exacerbating inequities in healthcare coverage and necessitating supplementary offline home-visiting services as a safeguard mechanism (36).

Although smart wearables and big-data-driven risk prediction technologies hold considerable promise, prospective validation studies specifically targeting postoperative pediatric cohorts remain scarce. Current evidence is largely derived from small-scale feasibility trials, which are insufficient to conclusively demonstrate reductions in long-term complications or improvements in quality of life (37). The algorithmic stability, data security, and clinical adaptability of these devices require substantiation through large-scale, multicenter studies. Until robust validation is achieved, routine deployment in the care of high-risk pediatric patients is not advisable. Importantly, the majority of studies supporting the efficacy of informatized nursing are single-center, non-randomized controlled trials characterized by limited sample sizes and a lack of blinding, thereby posing risks of selection and measurement biases. Furthermore, several studies rely exclusively on subjective caregiver-reported outcomes, compromising objectivity. Future research mandates rigorously designed multicenter randomized controlled trials (RCTs) to validate the long-term benefits of these interventions (38).

### 3.3 Multidisciplinary team (MDT)-based transitional care model

Postoperative rehabilitation for children with HD necessitates coordinated input from multiple disciplines, including pediatric surgery, gastroenterology, physical medicine and rehabilitation, clinical nutrition, and psychology. Given that individual nurses are seldom equipped to address these multifaceted needs in their entirety, the MDT-based transitional care model has been developed (39). Placing the child’s holistic recovery at its core, this model convenes a dedicated team comprising the surgical nurse manager, primary nurse, pediatric surgeon, rehabilitation therapist, clinical dietitian, psychologist, and community nurse, with clearly delineated roles and collaborative workflows (40).

Within this framework, the pediatric surgeon oversees postoperative evaluation, manages complications, and adjusts treatment regimens as clinically indicated. The rehabilitation therapist designs and supervises the implementation of protocols targeting anal sphincter training and intestinal rehabilitation. The clinical dietitian formulates individualized nutritional plans tailored to the child’s age, growth status, and gastrointestinal tolerance. The psychologist conducts periodic assessments of emotional well-being and delivers counseling alongside behavioral interventions. Complementing these efforts, the community nurse performs home visits and provides grassroots-level guidance, while the primary nurse assumes overall coordination—integrating multidisciplinary insights to dynamically refine the care plan (41).

The MDT model effectively transcends the limitations of unidimensional care by integrating disease management, functional rehabilitation, nutritional support, and psychosocial intervention. This approach has been shown to significantly improve recovery outcomes in patients with complex pathologies or prolonged disease courses (42), and is particularly indicated for high-risk pediatric populations, including those with long-segment or total colonic aganglionosis and those experiencing recurrent postoperative complications. While this model has been progressively implemented in tertiary pediatric specialty hospitals across China, its widespread adoption in primary care settings remains constrained by limited subspecialty resources and personnel allocations (43, 44). In contrast, developed nations in Europe and North America have incorporated MDT frameworks into standard postoperative HD care protocols. These teams typically comprise specialists from pediatric surgery, gastroenterology, and psychology, supported by dedicated care coordinators to ensure service continuity; such structures have yielded significant reductions in readmission rates among high-risk pediatric patients (39, 45). The 2025 European Reference Network for Rare Inherited and Congenital Anomalies (ERNICA) guidelines further stipulate that MDT teams must conduct joint evaluations for complex cases at three-month intervals to safeguard intervention coherence (39). Comparatively, in China, the MDT model remains largely confined to tertiary pediatric centers. Primary healthcare institutions face substantial barriers to replicating these models due to constraints in subspecialty configuration and healthcare reimbursement policies, underscoring the need for further refinement of cross-regional interdisciplinary collaboration mechanisms.

### 3.4 Three-tier linkage model of transitional care

The core of the three-tier linkage model lies in integrating the specialized resources of tertiary hospitals, primary community healthcare services, and family-based caregiving to establish a rehabilitation system characterized by clear hierarchies, well-defined responsibilities, and seamless connection. Tertiary hospitals are tasked with formulating standardized, individualized rehabilitation protocols, conducting targeted training for family caregivers and community nurses, and managing complicated postoperative complications alongside necessary care plan modifications (46). Community health service centers assume responsibility for routine follow-up within their jurisdictions, delivering fundamental rehabilitation guidance, supervising home-based care, and performing ongoing health monitoring (47). The home serves as the principal setting for implementing care; under professional guidance, families execute prescribed nursing measures and proactively report on the child’s recovery status (48).

This model effectively addresses systemic challenges, including excessive follow-up burdens in major hospitals, suboptimal professional competence at the primary care level, and inconsistent standards in home rehabilitation. By facilitating the downward extension of high-quality nursing resources, it expands both the coverage and sustainability of transitional care—proving particularly valuable for the long-term management of children residing in rural or geographically underserved areas (49). The UK’s National Health Service (NHS) has established an integrated “hospital-community-home” information platform that enables real-time data sharing for postoperative pediatric patients. This system allows community nurses to access electronic medical records from tertiary hospitals, while specialists can remotely review community follow-up documentation, thereby effectively standardizing grassroots care protocols (50, 51). In contrast, China’s healthcare institutions remain hampered by information silos across different administrative levels. Moreover, the general lack of specialized pediatric surgical training among community nurses has significantly compromised the implementation efficacy of the three-tiered linkage model. Persistent challenges include inadequate coordination mechanisms, poor vertical information exchange, insufficient professional competence within the community nursing workforce, and the absence of robust performance evaluation systems. Consequently, a standardized operational framework has yet to be fully established and warrants further optimization.

### 3.5 Precision individualized stratified nursing model

With the widespread adoption of precision nursing, stratified and individualized continuity of care tailored to the heterogeneous characteristics of children with HD has emerged as a prominent research focus. Departing from the conventional “one-size-fits-all” approach, this model categorizes patients into three risk strata—low-risk, intermediate-risk, and high-risk—based on comprehensive indicators including surgical procedure, length of aganglionic bowel segment, age at surgery, postoperative stage, risk level of complications, and family caregiving capacity. Differentiated interventions are then implemented accordingly: ① Low-risk patients(short-segment HD, favorable postoperative recovery, low incidence of complications): Management primarily consists of routine follow-up, health education, and general guidance, with appropriately reduced visit frequency and streamlined intervention content (52, 53). ② Intermediate-risk patients (classic-type HD, delayed recovery of bowel function, mild defecatory disturbances): Care emphasizes reinforced rehabilitation training, dietary modulation, and regular functional assessments, supported by increased follow-up frequency (54). ③ High-risk patients (long-segment or total colonic aganglionosis, recurrent HAEC, anal stricture, severe defecatory dysfunction): A MDT approach is initiated, featuring one-on-one case management, dynamic monitoring, timely protocol adjustment, and prioritized prevention of severe complications (55).

Within the stratified assessment framework, parental educational level should be regarded as a core determinant of family caregiving capacity. For caregivers with lower educational attainment, even pediatric patients categorized as low-risk require intensified follow-up frequency, simplified written health education materials, and reinforced competency assessments through practical demonstrations. This proactive approach mitigates the risk of disease progression due to caregivers’ inadequate comprehension. Conversely, families with higher educational levels may warrant reduced time allocation for basic care instruction; instead, educational efforts should prioritize the recognition of severe complications and long-term self-management strategies, thereby enabling the precise allocation of nursing resources. Furthermore, targeted empowerment training tailored to variations in caregivers’ literacy and competency levels facilitates a model characterized by patient stratification, nursing gradation, and precision intervention. This strategy significantly enhances both the utilization efficiency of nursing resources and the effectiveness of interventions, representing a pivotal direction for the future development of precision-oriented continuous care for HD (Table 2).

**Table 2:** Models of postoperative continuity of care for HD.

Nursing model	Follow-up format	Strengths	Limitations	Target population/Clinical scenario	Reference
Traditional follow-up	Telephone follow-up, outpatient revisit, home visit	Simple to implement; low cost; easy accessibility	Homogeneous content; poor interactivity; lack of systematic planning	Primary care hospitals; low-risk patients with mild disease	(23)
Informatization-based smart care	WeChat, mobile applications, tele-video, intelligent platforms	Round-the-clock service; real-time guidance; traceable data; improved adherence	Dependent on internet access and smart devices; limited adaptability in remote areas	Families with adequate digital access and device availability	(28–30)
MDT	Joint services by surgery, rehabilitation, nutrition, psychology, and nursing teams	Comprehensive, multidimensional intervention; addresses complex rehabilitation needs	Resource-intensive; difficult to implement in primary care settings	Long-segment/total colonic HD; high-risk patients with recurrent complications	(39, 40)
Hospital–community–family three-tier linkage	Hospital coordination, community implementation, family execution	Resource decentralization; broad coverage; alleviates tertiary hospital burden	Imperfect linkage mechanisms; insufficient professional expertise at community level	Long-term rehabilitation management in primary care and remote regions	(46, 49)
Precision individualized stratified nursing	Risk-stratified intervention based on disease severity, complication risk, and caregiving capacity	High resource utilization efficiency; strong intervention specificity	Requires well-defined risk-stratification criteria; heavy initial assessment workload	All-age HD patients, particularly those with heterogeneous risk profiles	(50, 51, 55)

## 4 Core components of postoperative continuity of care

### 4.1 Precision prevention and management of postoperative complications

The cornerstone of continuity of care for HD lies in the effective prevention and control of postoperative complications. Current research has established a comprehensive “early screening–dynamic monitoring–precision intervention–recurrence prevention” framework, with targeted strategies focusing on three major complications: HAEC, anal stenosis, and defecatory dysfunction.

HAEC is a critical postoperative complication of HD, clinically manifesting as fever, abdominal distension, diarrhea, and foul-smelling watery stools. Severe cases may precipitate septic shock or intestinal perforation (56). Recent studies emphasize preventive intervention strategies, including structured discharge education to ensure caregivers can recognize early warning signs and implement home monitoring focused on body temperature, abdominal girth, stool consistency, and defecation frequency (57). For high-risk patients, nursing protocols incorporate regular gut microbiota surveillance and caregiver guidance regarding standardized probiotic administration and home rectal irrigation. These measures aim to minimize fecal stasis and bacterial proliferation, thereby reducing HAEC incidence (58). Mild cases may be managed conservatively at home under telemedicine guidance, involving fasting, fluid repletion, bowel irrigation, and targeted anti-infective therapy to prevent deterioration. Conversely, severe cases necessitate immediate activation of a fast-track admission pathway to expedite inpatient care. Current research on HAEC prevention predominantly comprises retrospective cohort studies. Consequently, recommendations regarding gut microbiota modulation and rectal irrigation remain at a moderate evidence level, highlighting a critical need for large-scale prospective studies to define optimal intervention timing and standardize operational protocols (59).

Anal stenosis typically results from anastomotic scar hyperplasia or anal sphincter spasm, presenting with straining, narrowed stools, and dyschezia (60). A standardized protocol recommends initiating regular anal dilation 2 weeks postoperatively. Graded dilation regimens are tailored to the child’s age and anal tolerance, with nurses providing hands-on instruction to caregivers regarding technique, frequency, and depth. Home-based dilation is maintained for 3–6 months, complemented by regular clinical reassessments to adjust protocols dynamically (61). For refractory sphincter spasm, adjunctive interventions such as botulinum toxin injection (52, 53) and pelvic floor electrical stimulation may be employed to relieve spasticity and improve anal compliance.

Defecatory dysfunction—encompassing incontinence, constipation, and diarrhea—is the most prevalent long-term functional complication (62). Emerging interventions prioritize pelvic floor rehabilitation through staged pelvic muscle training, rectal sensory conditioning, and bowel habit retraining to restore continence and normal colonic motility (63). Diarrheal episodes are managed with antidiarrheal agents and low-volume enemas, accompanied by dietary modification. Refractory constipation is addressed through increased dietary fiber, abdominal massage, probiotic therapy, and scheduled toileting routines to optimize bowel function and prevent recurrence (64) (Table 3).

**Table 3:** Identification and nursing priorities for major postoperative complications.

Complication	Clinical manifestations	Nursing priorities	Interventions	Reference
HAEC	Fever, abdominal distension, foul-smelling watery diarrhea	Gut microbiota modulation; standardized rectal irrigation; dietary control; caregiver education on early symptoms	Mild: home-based fasting, hydration, irrigation, anti-infective therapy. Severe: immediate hospitalization via emergency pathway	(94)
Anal stenosis	Straining, narrow-caliber stools, difficult defecation	Initiate dilation at postoperative week 2; graded protocol based on age	Pelvic floor electrical stimulation; botulinum toxin injection; dynamic protocol adjustment	(52, 53, 95)
Defecatory dysfunction	Poor continence, chronic constipation, recurrent diarrhea	Staged pelvic floor training; toilet habit training; dietary optimization	Pharmacologic management; abdominal massage; probiotics; restoration of defecation rhythm	(64, 96)

### 4.2 Individualized nutritional support and growth surveillance

Preoperative chronic abdominal distension and poor intake, compounded by incomplete postoperative intestinal recovery, predispose HD children to malnutrition, underweight status, growth retardation, and anemia (65). Nutritional support and growth monitoring thus constitute essential elements of continuity care. Within MDT-based models, clinical dietitians formulate phased nutritional regimens: ① Early postoperative phase (first month after discharge): Emphasis on breast milk or formula, light, digestible, low-fat, and low-residue diets to minimize intestinal burden and prevent bloating or diarrhea (66). ② Intermediate phase (1–6 months): Gradual reintroduction of high-quality protein, dietary fiber, and micronutrients, progressing toward normalized eating patterns while avoiding overeating (67). ③ Beyond 6 months: Balanced diets tailored to growth status to correct deficiencies and promote catch-up growth.

A dynamic growth-monitoring archive is established, tracking height, weight, head circumference, hemoglobin, and serum albumin against age-appropriate norms. Early identification of growth faltering triggers targeted dietary adjustments or supplemental nutrition. Severely malnourished patients receive oral nutritional supplements and micronutrient repletion to optimize outcomes. Systematic, individualized nutritional continuity care significantly improves long-term growth prognosis.

### 4.3 Psychosocial and behavioral intervention

Historically, postoperative HD care focused predominantly on somatic recovery; however, psychological well-being has gained increasing attention. Chronic defecatory disturbances, repeated hospitalizations, and prolonged home recuperation render children vulnerable to anxiety, low self-esteem, social withdrawal, irritability, and behavioral problems (68). School-aged children are particularly prone to peer relationship difficulties, school avoidance, and diminished self-confidence.

Modern continuous care models incorporate systematic psychological intervention as a core component. Specialized professionals conduct regular assessments of pediatric patients’ psychological status. For younger children, play therapy, companionship, and reassurance are employed to alleviate irritability. School-aged children benefit from psychological counseling, positive reinforcement, and parental empowerment strategies designed to foster a realistic understanding of their condition, encourage participation in group activities, and enhance social adaptability (69). Concurrently, the care team provides family-centered psychological support targeting caregivers experiencing anxiety or excessive burden. This approach alleviates caregiving stress, cultivates a home environment conducive to recovery, and indirectly improves the child’s psychological wellbeing (70). Additionally, behavioral modification programs are implemented to correct maladaptive habits stemming from chronic defecation disorders, promoting regular routines and scheduled bowel habits to comprehensively improve overall physical and mental health. Crucially, parental educational attainment moderates the efficacy of psychological support delivery. Highly educated parents are better equipped to comprehend child developmental psychology and collaborate effectively with clinical staff. Conversely, parents with lower educational levels may overlook subtle emotional changes due to cognitive constraints or inadvertently transmit their own anxiety to the child (71). Therefore, psychological interventions must integrate cognitive guidance for parents to elevate the overall supportive capacity of the family unit.

### 4.4 Family empowerment and health management

Family caregiving quality is a decisive determinant of rehabilitation success; therefore, caregiver empowerment forms the backbone of continuity care (72). Current programs integrate structured training modules, including pre-discharge workshops, one-on-one skills coaching, ongoing online education, troubleshooting clinics, and competency assessments. Pre-discharge sessions emphasize mastery of anal dilation, rectal irrigation, complication recognition, dietary management, and emergency response, reinforced through demonstration, supervised practice, and corrective feedback (61). Post-discharge, video-based tutorials and home visits sustain skill retention and rectify improper techniques. Regular dissemination of updated knowledge and real-time problem solving further consolidates caregiver competence. Periodic evaluations identify gaps and guide targeted remediation (73).

Furthermore, the continuous care team prioritizes enhancing caregivers’ disease management awareness and fostering a mindset oriented toward long-term rehabilitation. This approach mitigates the risk of nonadherence—such as prematurely discontinuing rehabilitation training or scheduled follow-ups upon short-term symptomatic relief—thereby significantly improving postoperative long-term compliance. Consequently, a virtuous rehabilitation cycle characterized by “professional guidance coupled with efficient family implementation” is established. Caregiver empowerment must account for the heterogeneity in parental educational attainment (74). For parents with lower educational levels, communication strategies should avoid medical jargon; instead, local dialect explanations, animated demonstrations, and repeated competency assessments should be employed to reinforce retention. Conversely, for highly educated parents, providing access to authoritative literature and advanced nursing knowledge can facilitate their informed participation in rehabilitation decision-making, while preventing unnecessary anxiety induced by information overload.

### 4.5 Long-term follow-up and dynamic prognostic management

Given the protracted recovery trajectory, some HD patients may develop late-onset defecatory dysfunction or chronic intestinal inflammation, underscoring the necessity of lifelong surveillance (75). Structured follow-up schedules include early post-discharge assessments to detect acute complications, periodic evaluation of anastomotic healing and bowel function, and milestone visits at 6 months, 1, 2, and 3 years to monitor defecatory status, growth, and psychosocial adaptation (76). For high-risk phenotypes—such as total colonic or long-segment aganglionosis—permanent medical records and lifelong health management plans are advised (77). Follow-up has evolved beyond biomedical parameters to encompass multidimensional appraisal spanning somatic function, psychological wellbeing, growth, quality of life, and social integration (78). Continuous feedback loops enable iterative refinement of rehabilitation strategies, minimizing adverse events and maximizing long-term prognosis (56).

## 5 Evaluation system for continuity of care

Current research on nursing outcome evaluation systems exhibits significant heterogeneity. Substantial variations exist across studies regarding the selection of assessment scales, scheduling of follow-up timepoints, and definitions of outcome measures, thereby hindering cross-study comparisons. Furthermore, most studies fail to adjust for confounding variables—such as parental educational attainment and household socioeconomic status—which risks overestimating the isolated efficacy of continuous care interventions. Consequently, the methodological quality of the existing evidence base requires substantial improvement. A scientifically rigorous and comprehensive evaluation framework is essential for ensuring quality control and refining intervention protocols. Current HD continuity-care metrics have progressed from isolated clinical endpoints to multidimensional composite systems comprising five domains: ① Clinical Outcomes: Incidence of postoperative complications, readmission rates, and time to wound healing or defecatory normalization provide objective evidence of safety and efficacy, representing the most widely adopted indicators (79). ② Bowel Rehabilitation: Validated instruments (e.g., Bowel Function Score (BFS)) combined with physiologic testing assess stool frequency, continence, sphincter performance, and motility, enhancing scientific rigor (80). ③ Psychosocial Dimensions: Standardized tools such as the Pediatric Quality of Life Inventory (PedsQL) (80) and the Pediatric Inventory for Parents/Coping Strategy Scales (81) quantify emotional health, social competence, and family quality of life, addressing prior somatic-centric biases. ④ Service Process Dimension: The evaluation framework has been expanded to incorporate novel indicators such as family caregiving capacity, adherence rates to rehabilitation regimens and scheduled follow-ups, caregivers’ disease-specific health literacy, and nursing satisfaction. This integration facilitates a dual-focused assessment system that evaluates both clinical outcomes and service processes (82). Crucially, within the assessment of caregiving capacity, parental educational attainment must be included in the statistical analysis as a key covariate. Adjusting for this variable mitigates its confounding effect on metrics including health literacy and adherence rates, thereby enabling a more precise quantification of the true efficacy of nursing interventions. ⑤ Health Economics: Comparative analyses of direct medical costs, length of stay, and readmission expenditures substantiate the resource-conserving value of continuity care, strengthening its policy relevance. The evidence grades corresponding to the various core nursing interventions are summarized in Table 4.

**Table 4:** Summary of evidence grades for core interventions in continuous care following HD Surgery.

Core nursing intervention category	Specific intervention measures	Level of evidence	Brief description of the evidence
Precise prevention and control of postoperative complications nursing	HAEC home monitoring, preventive intestinal irrigation, probiotics to regulate intestinal flora	Level III	Multicenter retrospective cohort studies have confirmed the ability to reduce the incidence of enteritis, but there is a lack of large-sample prospective RCTs to verify the optimal operational norms.
	Standardized graded dilation after surgery, pelvic floor electromyography stimulation to improve anal stenosis	Level I	Domestic and international guidelines, meta-analyses unanimously recommend, and high-quality controlled research evidence is sufficient.
	Pelvic floor muscle training, defecation behavior correction to improve incontinence/constipation	Level II	Single-center RCTs have confirmed short-term improvement in defecation function, but long-term quality of life evidence still needs to be supplemented.
Individualized nutrition and growth development care	Staged and stepwise dietary management, regular dynamic monitoring of growth indicators	Level II	Cohort studies have confirmed the ability to improve the nutritional status of children, but there is still a lack of a unified standardized nutritional intervention plan.
Psychological behavior and family care empowerment	Age-appropriate games/communication psychological counseling, caregiver practical training + multimodal education	Level IV	Most studies are cross-sectional and qualitative, with small sample size intervention trials being effective, but lacking long-term follow-up RCTs.
Mainstream nursing model and follow-up management	Traditional telephone/clinic follow-up	Level III	Basic routine interventions have been proven by a large number of clinical studies to be able to prevent and control complications.
	Informationized smart online nursing (WeChat/APP/remote video)	Level II	Single-center RCTs have demonstrated improving compliance and shortening the recovery period, but the evidence of adaptability for remote areas is insufficient.
	MDT multidisciplinary collaborative care	Level III	The inclusion criteria of the European and American HD guidelines, and the cohort studies of tertiary hospitals have improved the prognosis of high-risk children.
	Hospital-community-family three-level linkage care	Level IV	Only regional pilot studies have been conducted, and the evidence of the effectiveness of implementation at the grassroots level is limited, and the information sharing gap is obvious.
	Precise stratification and individualized care	Level IV	The feasibility trial of risk stratification models is effective, but the unified stratification standards have not yet been formed.
Long-term follow-up and prognosis management	Periodic follow-up at different stages, lifelong electronic health record tracking	Level III	The consensus of domestic and international experts is mandatory recommendation, and long-term cohort studies have confirmed the ability to identify delayed complications early.

Evidence grading was performed in accordance with the criteria proposed by Vatkar A et al. (97): Level I, systematic reviews and meta-analyses; Level II, RCTs; Level III, cohort and case–control studies; Level IV, case series and case reports; Level V, expert opinion and anecdotal evidence.

## 6 Limitations and unmet needs in current research

Current research on transitional care following HD surgery in children has achieved certain progress; however, several limitations persist, hindering the standardization, normalization, and widespread implementation of nursing services (61). To date, no unified postoperative transitional care guidelines or standardized protocols have been established internationally or domestically. Significant heterogeneity exists across institutions regarding care models, intervention components, follow-up frequencies, and rehabilitation regimens (83). Moreover, most existing studies are single-center, small-sample observational designs, resulting in poor generalizability and reproducibility. This undermines the development of unified industry standards and leads to suboptimal care homogeneity (84).

Informatization and intelligent nursing technologies remain at a rudimentary stage of application, primarily limited to online health education, follow-up reminders, and basic clinician-patient interaction. The development of advanced functionalities—such as big data- and artificial intelligence-driven risk stratification, prognostic prediction, and intelligent interventions—is lacking, preventing the full realization of smart nursing’s technical advantages. Furthermore, the adoption of smart devices and online services is constrained by geographic and socioeconomic disparities, limiting their coverage and accessibility in primary care settings and remote regions (5). Current studies generally suffer from short follow-up durations, focusing predominantly on short-term postoperative outcomes within the first 6–12 months. Long-term investigations into long-term prognosis, pubertal growth and development, lifelong quality of life, and delayed complications remain scarce. Specialized intervention studies targeting high-risk subgroups—such as total colonic aganglionosis, neonatal surgeries, and patients with comorbidities—are also insufficient, underscoring the need for refined precision intervention frameworks (85).

Multidisciplinary collaboration and the tertiary linkage model integrating hospitals, communities, and families have only been implemented in select medical centers. Primary care institutions face constraints related to disciplinary configuration, staffing, and funding, impeding the delivery of systematic services. Poor information interoperability, ambiguous role delineation, and gaps in referral continuity further compromise care quality, while inadequate professional competence among community nurses contributes to service fragmentation and inconsistent standards. Additionally, current nursing practice remains centered on physical recovery and complication prevention. Research on psychological-behavioral interventions and social adaptation training is still nascent, characterized by fragmented protocols and a lack of age-specific standardized psychological intervention workflows and quantitative evaluation systems. Long-term psychological monitoring and support mechanisms for both patients and caregivers require further refinement (86). Finally, prevalent sociodemographic factors—particularly parental educational level—are often inadequately addressed. Most studies fail to include educational level in baseline equilibrium tests or as a stratification variable to analyze differential nursing benefits across educational strata (87, 88). This oversight risks selection bias and may lead to over- or underestimation of intervention effects, highlighting a critical gap to be addressed in future research.

## 7 Future perspectives

Building on current research achievements and addressing existing gaps, transitional care following pediatric HD surgery is poised to evolve toward greater standardization, precision, intelligence, continuity, and diversification. First, multicenter, large-sample, long-term evidence-based studies must be conducted to develop unified clinical guidelines and standardized protocols tailored to domestic practice. Stratified care pathways should be established based on disease severity, surgical approach, and patient age to promote nationwide care homogeneity (89).

Second, deeper integration of big data, artificial intelligence (AI), the Internet of Things (IoT), and wearable devices into nursing practice is essential (77). However, smart devices remain in the clinical exploratory stage; no dedicated algorithmic models specifically adapted to the HD postoperative recovery context currently exist, and the risk-prediction capabilities of most intelligent tools lack external validation. Future efforts should prioritize prospective cohort studies to delineate the applicability, efficacy boundaries, and ethical frameworks governing these technologies, thereby preventing unjustified large-scale deployment. Concurrently, primary care–oriented intelligent service models should be optimized by developing low-digital-barrier assistive tools to expand coverage and narrow urban–rural disparities in nursing resources.

For high-risk subgroups—including total colonic aganglionosis, long-segment HD, neonatal cases, and patients with recurrent postoperative complications—targeted investigations are warranted to construct robust risk-stratification models. Individualized, “one-patient-one-plan” interventions should be implemented based on comprehensive patient profiling to refine precision nursing systems for vulnerable populations (90). Parental educational level must be incorporated as a core dimension in risk-stratification models. Parallel development of educational tools and intervention pathways adapted to varying literacy levels is critical: for families with lower educational attainment, visual, colloquial, and hands-on instructional materials should be promoted alongside regular home visits by community nurses; for highly educated families, structured knowledge repositories and self-management tools should be provided to enhance precision and relevance.

Furthermore, long-term cohort studies are needed to track bowel function, growth and development, psychological status, and late-onset complications across years, through puberty, and into adulthood (75). Establishing lifelong electronic health records will enable dynamic, whole-life-cycle health management models. Additionally, multidisciplinary collaboration workflows must be streamlined with clear role delineation to extend MDT models to primary care settings (91). The tertiary linkage mechanism integrating hospitals, communities, and families should be strengthened through shared information platforms and enhanced specialist training for community nurses, eliminating barriers between service tiers (7).

**Figure 1:**
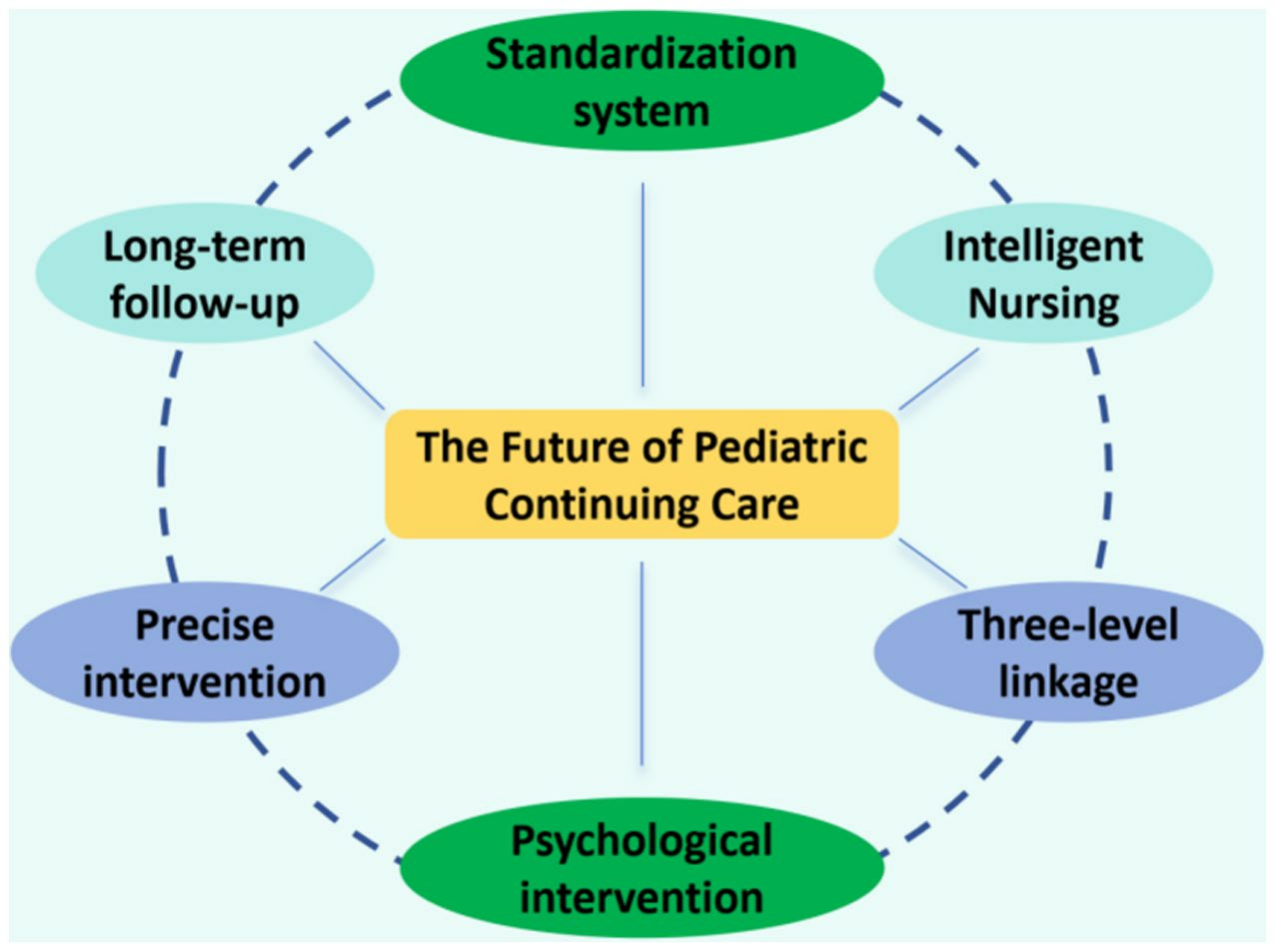
Future development direction of continuity care after HD surgery.

Finally, research on psychological and behavioral interventions must be intensified. Age-specific standardized protocols and evaluation metrics should be established, incorporating psychological counseling and social skills training into routine care. Family-centered psychosocial support and caregiver empowerment systems require further refinement, with particular attention to how parental educational level moderates intervention outcomes. Differentiated psychological support plans should be designed for families with varying cognitive capacities to build a collaborative rehabilitation network that comprehensively enhances children’s physical and mental recovery and long-term quality of life. Drawing on mature international transitional care models, dedicated pediatric-to-adult care transition pathways for adolescent HD patients should be instituted to address gaps in long-term follow-up and meet the demands of lifelong health management (Figure 1).

## 8 Conclusion

Continuity of care following surgical intervention for pediatric HD constitutes a cornerstone for accelerating postoperative recovery, mitigating long-term complications, and enhancing lifelong prognosis. Driven by advancements in nursing philosophy and medical technology, continuity of care for these patients has evolved from conventional single-modality telephone follow-ups into a multifaceted system characterized by informatization, multidisciplinary collaboration, stratified precision interventions, and a three-tier linkage model integrating hospitals, communities, and families. The scope of interventions now encompasses multiple dimensions, including complication surveillance, bowel function rehabilitation, and individualized nutritional support. Concurrently, the supporting evaluation frameworks have been progressively refined, providing robust infrastructure for the sustained recovery of affected children. Nevertheless, several critical challenges persist within this field: the absence of standardized protocols, insufficient depth regarding intelligent application deployment, a paucity of long-term follow-up studies, inadequate primary care capacity, and underdeveloped psychological intervention systems. Of particular note, parental educational level—a key sociodemographic determinant—significantly influences family caregiving competence, treatment adherence, and, ultimately, patient outcomes. Consequently, future investigations must incorporate this variable as a core consideration in study design and data analysis to mitigate confounding bias and bolster the scientific rigor of the resultant evidence base. Future endeavors should prioritize advancing continuity of care toward greater standardization, precision, intelligence, and whole-course management. This entails refining targeted intervention systems for high-risk pediatric populations, establishing comprehensive life-cycle health management models, optimizing multidisciplinary collaboration and three-tier linkage mechanisms, and elevating overall nursing quality to promote the long-term physical and mental health and quality of life of children with HD.
